# Daily Stressors Degrade Perceived Cognitive Abilities in Healthcare Professionals

**DOI:** 10.1093/geroni/igaa057.3289

**Published:** 2020-12-16

**Authors:** Rhitik Joshi Taylor Drury, Soomi Lee

**Affiliations:** University of South Florida, Tampa, Florida, United States

## Abstract

Stress negatively impacts cognitive functioning. Less is known about whether daily stress is associated with perceived cognitive abilities in healthcare workers who require mental sharpness and attention to provide high-quality patient care. We examined daily associations between stressors and perceived cognitive abilities in nurses and whether the associations differed between workday vs. non-workday. Using 14-day smartphone-based ecological momentary assessment, 61 inpatient nurses at a U.S. cancer hospital reported the frequency and severity of daily stressors (e.g., arguments, accidents). Each day, participants subjectively evaluated their mental focus, memory, and attention. Multilevel modeling examined the within- and between-person associations of daily stressors with cognitive abilities adjusting for sociodemographics, work shift, and workday. Nurses reported experiencing stressors once every other day. More stressors were associated with poorer cognitive abilities. At the between-person level, those with more frequent or severe stressors reported poorer mental focus (B=-22.4, p<.01; B=-0.35, p<.01, respectively), worse memory (B=-24.35, p<.01; B=-0.37, p<.01, respectively), and lower attention (B=-25.47, p<.05; B=-0.40, p<.01, respectively). At the within-person level, on days with more frequent or severe stressors, participants reported poorer mental focus (B=-2.05, p<.05; B=-.03, p<.05, respectively) and lower attention (B=-1.95, p<.05; B=-.04, p<.01, respectively). Some of the between-person associations were more apparent on workdays; those with more stressors reported poorer mental focus and lower attention on workdays than on non-workdays. Nurses’ perceived cognitive abilities at work vary by daily stressors. Disconnecting the linkage between stressors and perceived cognition may help improve work performance in nurses who may encounter frequent stressors at work.

